# The Role of Disease Severity and Demographics in the Clinical Course of COVID-19 Patients Treated With Convalescent Plasma

**DOI:** 10.3389/fmed.2021.707895

**Published:** 2022-01-26

**Authors:** Tengfei Ma, Chad C. Wiggins, Breanna M. Kornatowski, Ra'ed S. Hailat, Andrew J. Clayburn, Winston L. Guo, Patrick W. Johnson, Jonathon W. Senefeld, Stephen A. Klassen, Sarah E. Baker, Katelyn A. Bruno, DeLisa Fairweather, R. Scott Wright, Rickey E. Carter, Chenxi Li, Michael J. Joyner, Nigel S. Paneth

**Affiliations:** ^1^Department of Epidemiology and Biostatistics, College of Human Medicine, Michigan State University, East Lansing, MI, United States; ^2^Department of Anesthesiology and Perioperative Medicine, Mayo Clinic, Rochester, MN, United States; ^3^Department of Quantitative Health Sciences, Mayo Clinic, Jacksonville, FL, United States; ^4^Department of Cardiovascular Medicine, Mayo Clinic, Jacksonville, FL, United States; ^5^Department of Cardiovascular Medicine and Director Human Research Protection Program, Mayo Clinic, Rochester, MN, United States; ^6^Department of Pediatrics and Human Development, College of Human Medicine, Michigan State University, East Lansing, MI, United States

**Keywords:** convalescent plasma therapy, antibody therapy, SARS-CoV-2, COVID-19, antibodies

## Abstract

Treatment of patients with COVID-19 using convalescent plasma from recently recovered patients has been shown to be safe, but the time course of change in clinical status following plasma transfusion in relation to baseline disease severity has not yet been described. We analyzed short, descriptive daily reports of patient status in 7,180 hospitalized recipients of COVID-19 convalescent plasma in the Mayo Clinic Expanded Access Program. We assessed, from the day following transfusion, whether the patient was categorized by his or her physician as better, worse or unchanged compared to the day before, and whether, on the reporting day, the patient received mechanical ventilation, was in the ICU, had died or had been discharged. Most patients improved following transfusion, but clinical improvement was most notable in mild to moderately ill patients. Patients classified as severely ill upon enrollment improved, but not as rapidly, while patients classified as critically ill/end-stage and patients on ventilators showed worsening of disease status even after treatment with convalescent plasma. Patients age 80 and over showed little or no clinical improvement following transfusion. Clinical status at the time of convalescent plasma treatment and age appear to be the primary factors in determining the therapeutic effectiveness of COVID-19 convalescent plasma among hospitalized patients.

## Introduction

The number of deaths from COVID-19 in the United States (US) had surpassed 500,000 by February 11, 2021 (1), less than a year after the first case of novel coronavirus (SARS-CoV-2) was confirmed in the US, demonstrating the urgent need to find safe and effective treatment options. Convalescent plasma, rich in antibodies from recently recovered patients, was used successfully in the 1918 influenza pandemic (2), SARS-1 (3), and Ebola (4) epidemics. Recognizing that a vaccine would not be widely available for several months to a year, and facing a paucity of treatment options, the US Federal Government, in collaboration with the Mayo Clinic and the national blood banking community, developed the Expanded Access Program (EAP) for COVID-19 convalescent plasma as a national registry to provide access to this potentially life-saving treatment, examine the safety, and as much as possible, the efficacy of convalescent plasma treatment in hospitalized patients. The inclusion criteria of the EAP required that enrolled patients (1) have a laboratory-confirmed diagnosis of COVID-19, (2) be severely ill or at high risk for becoming severely ill from COVID-19, and (3) be admitted to an acute care facility for COVID-19 complications.

Although the data are not fully consistent, suggestions of efficacy of convalescent plasma have emerged from retrospective comparisons of treated and untreated patients (5), from several randomized trials (6–15), and from analyses showing mortality in transfused patients is related in dose-response fashion to antibody titer in the plasma (10, 16). However, few studies in hospitalized patients have stratified patients based on disease severity at time of treatment to examine efficacy. Analyses of the EAP so far have shown that COVID-19 convalescent plasma is safe (17, 18) and likely to be effective at treating COVID-19, if antibody titers are sufficiently high (16). Based on the clear findings in the historical convalescent plasma literature (19), we hypothesized that patients treated earlier in the course of the disease [who were not on mechanical ventilation or admitted to the intensive care unit (ICU)] or who had less severe disease at the time of transfusion would show more rapid and better improvement than convalescent plasma recipients receiving mechanical ventilation or admitted to the ICU.

## Methods

### Study Design

The Mayo Clinic Expanded Access Program (EAP) was a national, multicenter, open-label registry of hospitalized adults with severe/life-threatening or at high risk of developing severe COVID-19 disease. This program allowed physician access to convalescent plasma as treatment for COVID-19 prior to the issuance of an emergency use authorization by the FDA. The initiation and approval of the program have been described in detail (17, 18). Briefly, hospital and physician registration occurred through the EAP central website, www.uscovidplasma.org. The web-based registration, compliance, and data entry system for the EAP went live on April 3, 2020, and the first transfusion was given on April 7, 2020. Written informed consent was obtained from the patient or legally authorized representative prior to enrollment, except for those patients who used an emergency consent process defined in collaboration with the US Food and Drug Administration (21 CFR 50.23). The study was approved and overseen by the Mayo Clinic Institutional Review Board (IRB #20-003312).

### The Rapid Evaluation Project (REP)

Because the EAP was developed primarily as a registry to investigate the safety of convalescent plasma as a treatment for COVID-19, during an ongoing pandemic and implemented during a time with limited clinical research resources, including hospital restrictions to essential personnel, research staff assigned other clinical duties, we developed short report forms to overcome these challenges. Also, because many in the health system were overwhelmed during the time of the EAP, we developed the REP to be an optional reporting tool that required minimal time and effort on the part of the treating physician, but that would, nonetheless, provide vital information on whether improvement or worsening was noted following treatment with convalescent plasma, and how this varied by category of patient.

We offered all physicians enrolling patients in the EAP the opportunity to participate in the Rapid Evaluation Project, by providing brief, daily updates of the status of their patients until death or discharge from the hospital. Physicians or their designees, provided the baseline status (mild/moderate, OR severe, OR critical, OR end-stage) of each participant on the day of enrollment. Each day after enrollment physicians or their designees were asked to complete a status update on the patient where they selected one of the following describing each participant's status compared to the previous day—discharged from hospital, better, the same, worse, or died, and the physician also noted whether the patient was in an ICU or had required mechanical ventilation on each reporting day.

This simple system was adapted from the study by Waller and Lawther who asked London patients with chronic obstructive pulmonary disease to record their daily status—‘better, worse, much worse, or the same as usual', and used these scores to document a clear relationship of worse days to specific components of air pollution (20, 21).

The Rapid Evaluation Project (REP) was an optional sub-study of the EAP, first implemented on May 5, 2020. Physicians or their designees who opted to participate were asked to answer three questions on a daily basis. Two questions were about the patient's status that day—

1. Has the patient been in the ICU in the past 24 h?
2. Has the patient required mechanical ventilation in the past 24 h?
3. The third question asked how the patient's status changed in the last 24 h? Possible answers to question 3 were:
  - The patient was discharged from hospital,
  - The patient improved
  - the patient stayed the same
  - the patient worsened
  - the patient died.

Physicians and/or their designee received an automatic daily notification by email requesting the status update. Participant enrollment in the REP was open to any patient [patient inclusion/exclusion criteria for EAP described previously (17, 18)] in the EAP whose physician or designee were willing to provide daily updates.

### Data Analysis

To define each patient's overall level of improvement or worsening on each day of observation, we calculated the total number of “condition worsened” and “condition improved” responses for each patient for the days preceding the day of observation. We used a 5-point ordinal scale to quantify these clinical outcomes as follows: −2 points, death; −1 point, clinical worsening; 0 points, no change; 1 point, clinical improvement; 2 points, discharge. If a participant had more “condition worsened” than “condition improved” responses, but was still alive and still hospitalized, we defined the overall clinical outcome as clinical worsening (−1) for that day. If the participant had a greater number of “condition improved” responses, while still hospitalized, we defined the overall clinical outcome as clinical improvement (+1) for that day. If the participant had the same total number of “condition worsened” and “condition improved” responses, but still hospitalized, we defined the overall clinical outcome as no change (0). Finally, death and discharge were also considered clinical outcomes giving us five potential clinical outcomes. To plot group trajectories, we averaged the ordinal scales of the patient group for each day of observation. To account for the status of all patients, including those discharged or died, patients were scored as either +2 or −2 on each day from their discharge or death until day 21.

We performed a linear segmented analysis to identify the changes of scores over time in each trajectory (22–24). This method allowed us to test for significant increasing or decreasing linear trends in clinical outcomes after transfusion. We set one breakpoint for each segmented regression model to determine if there was a point at which the trajectory for the individual materially changed. R package “segmented” was used for this data analysis (22, 23) and *P* < 0.001 was considered statistically significant.

#### Multivariate Analysis

A generalized estimating equations (GEE) approach with a logit link and independence “working” correlation structure was used to study the effect of age, sex, level of disease severity, baseline ventilator use, and baseline ICU admission when the transfusion was administered. The model included the clinical status (worsening/improvement) defined above on days 7, 14, and 21 as repeated outcome measures. Patients who died or were discharged on days other than day 7, 14, or 21 were considered deceased or discharged on the next interval. For example, a patient who died on day 8 would be categorized as deceased on day 14. To model the effect of baseline level of severity on different time points, the regression model included a time-varying term for baseline level of severity. All statistical analyses were completed using R version 4.0.2 (25). R package “multgee” was used for GEE analysis of ordinal multinomial responses (26, 27) and *P* < 0.05 was considered statistically significant.

## Results

### Patient Demographics and Baseline Severity

Sufficient follow-up information was submitted on 8,311 convalescent plasma recipients. Descriptions of the demographics and disease characteristics are presented in Table 1. Five hundred and thirty-four patients were excluded due to receipt of multiple transfusions during their hospitalization, and 597 patients who had <2 days of follow-up data, leaving a total of 7,180 patients for analysis. Participants in the REP were very similar to the overall EAP population at the time of data analysis (August 1, 2020) in age distribution, sex, race and initial clinical status, but tended to have slightly more respiratory risk factors such as dyspnea, low oxygen parameters and extensive early lung infiltrates. Compared to the EAP cohort (Table 1, third column), there were fewer Hispanic/Latino patients and more patients residing the Midwest and the West regions of the U.S., and fewer from the Northeast. Patients were severely ill with 53% in an ICU and 28% requiring mechanical ventilation prior to convalescent plasma transfusion.

**Table 1:** Demographic factors and disease severity in patients in the Rapid Evaluation Program and the overall expanded access program^†^ patients.

	**Rapid evaluation project sample^**†**^**	**EAP cohort^†^**
**Patient characteristics**		
Total patients enrolled	9,752	73,258
Enrolled patients that received a transfusion	8,311	64,987 (100.0%)^*^
Patients excluded due to insufficient follow-up^a^	381	–
Received >1 transfusion^b^	534	3,880 (6.0%)
Patients included in the analysis	7,164 (100%)^*^	–
**Patient outcomes**		
Discharge reported^c^	4,435 (60.0%)	44,926 (69.1%)
Death reported	1,659 (22.4%)	18,650 (28.7%)
**Geographic region**		
Midwest	1,473 (20.6%)	9,079 (14.0%)
Northeast	913 (12.7%)	11,410 (17.6%)
Puerto Rico	13 (0.2%)	67 (0.1%)
Southeast	2,252 (31.4%)	18.979 (29.2%)
Southwest	1,077 (15.0%)	16,161 (24.9%)
West	1,436 (20.0%)	9,289 (14.3%)
**Categorical age (years)**		
18–34	406 (5.7%)	3,661 (5.6%)
35–54	1,983 (27.7%)	17,236 (26.5%)
55–79	3,946 (55.1%)	36,682 (56.4%)
80 or older	829 (11.6%)	7,408 (11.4%)
**Gender**		
Female	2,872 (40.2%)	26,281 (40.5%)
Male	4,267 (59.7%)	38,472 (59.4%)
Undisclosed	7 (0.1%)	64 (0.1%)
**Race**		
Asian	251 (3.5%)	2,211 (3.4%)
Black or African American	1,423 (19.9%)	11,864 (18.3%)
Other or unknown	1,840 (25.7%)	17,170 (26.4%)
White	3,650 (50.9%)	33,742 (51.9%)
**Ethnicity**		
Hispanic/latino	2,494 (34.8%)	25,640 (39.5%)
Not hispanic/latino	4,670 (65.2%)	39,347 (60.5%)
**Initial clinical status**		
Mild or moderate	2,295 (34.0%)	4,196 (37.0%)
Severe	2,921 (43.3%)	4,741 (41.8%)
Critical or end stage	1,516 (22.5%)	2,370 (20.9%)
**Medications received during hospital stay**		
Angiotensin receptor blockers (ARBs)	455 (7.2%)	2,751 (6.7%)
ACE Inhibitors	584 (9.2%)	3,529 (8.6%)
Azithromycin	3,029 (47.7%)	20,043 (49.0%)
Remdesivir	2,823 (44.5%)	15,608 (38.2%)
Steroids	4,074 (64.2%)	26,843 (65.7%)
Hydroxychloroquine and/or chloroquine	646 (10.2%)	7,323 (17.9%)

† *All enrollment information as of August 3, 2020*.

* *Following subsections of the same column are based on this value*.

a *Insufficient follow-up include, no daily reporting for a patient, no dates of submitted reports*.

b *Defined as >4 h between transfusions. Multiple units given within 4 h were considered a single transfusion*.

c *Number of patients reported discharged or expired within 21 days of convalescent plasma treatment*.

### Score Trajectories Following Transfusion

**Figure 1:**
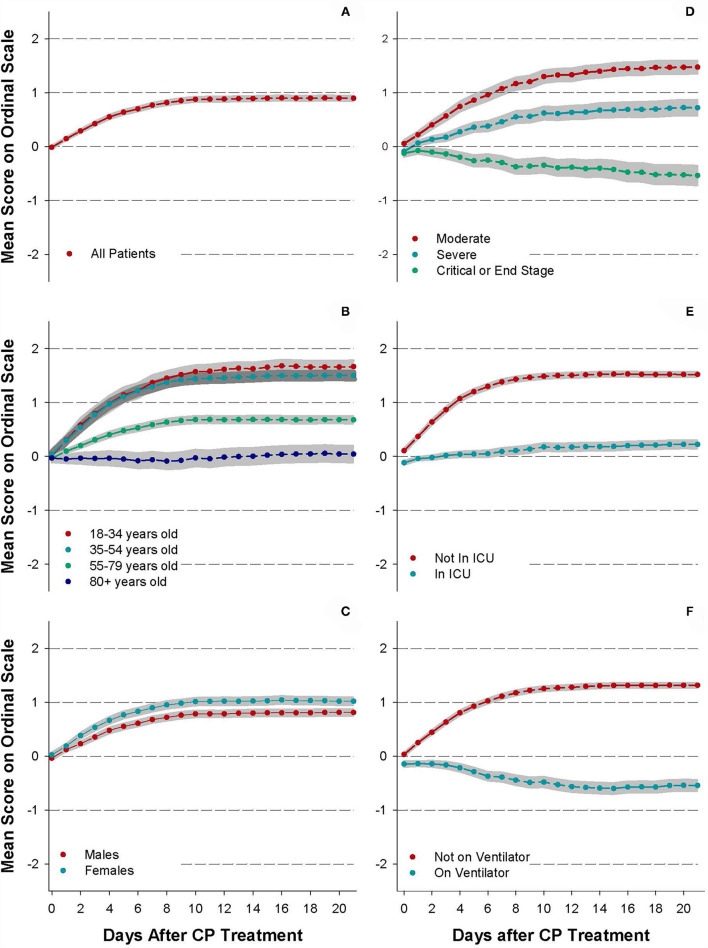
Trajectories of daily status improvement/worsening. Subgroup analyses include **(A)** all patients, **(B)** by age, **(C)** by sex, **(D)** by initial clinical status, **(E)** by ICU status prior to transfusion, and **(F)** by ventilator status prior to transfusion. Net patient scores of 0 = no net change, +1 = net improvement but still hospitalized, −1 = net worsened and still hospitalized, +2 = discharged from hospital, and −2 = patient expiration. Points are the mean score for each day, and gray bands indicate the 95% confidence interval for each data set.

Figure 1 displays the trajectories of mean ordinal scale scores for all patients (Figure 1A) and for several patient sub-groups (Figures 1B–F) for the first 21 days following CP transfusion.

For all patients in the REP (Figure 1A), the mean daily score increased rapidly from 1 day after CP infusion (mean = 0.16, 95% CI: 0.14–0.19) to day 8 (mean = 0.82, 95% CI: 0.78–0.87). Thereafter, the mean scores stabilized between 0.82 and 0.91.

When stratified by age group (Figure 1B), only the elderly (80+ years old) did not improve following transfusion but remained, on average, in the same status as at the time of transfusion 2 weeks following infusion (Day 14, mean = 0.002, 95% CI: −0.15, 0.15). While patients under 55 years of age improved more rapidly than patients 80 years and older, no substantial difference in improvement was seen between patients aged 18–34 years and patients aged 35–54 (Figure 1B). Overall, women tended to improve slightly more rapidly than did men, but both sexes exhibited overall improvement following treatment with convalescent plasma (Figure 1C).

When stratified by baseline category of illness severity (Figure 1D), patients with mild/moderate illness and severe illness improved most rapidly through day 7 and 9, respectively, and then leveled off. Patients described as critical/end-stage worsened from day 1 (mean = −0.04, 95% CI: −0.08, 0.01) onward following the transfusion to day 9 (mean = −0.26, 95% CI: −0.37, −0.16) and then slightly improved but with scores still remaining negative from day 9 to day 21 (mean = −0.19, 95% CI: −0.31, −0.08). Among all patients discharged from the hospital during the period of observation, the median length of stay following transfusion was 6 days (interquartile range = 4–11 days), and among patients who died, median length of stay following transfusion was 8 days (IQR = 4–13 days).

The leveling off of improvement after day 9 in the overall patient population (Figure 1A) appears to be attributable to patients with longer hospitalizations who may remain critically ill but medically stable; very long lengths of stay have been observed in some patients surviving with COVID-19 (28). Patients not in the ICU at the time of convalescent plasma treatment (Figure 1E) improved more steadily than patients in the ICU. Patients who did not require mechanical ventilation at the time of convalescent plasma treatment (Figure 1F) also had better improvement than patients requiring mechanical ventilation. Patients on ventilators declined steadily for 2 weeks, after convalescent plasma treatment (mean = −0.59, 95% CI: −0.69, −0.50).

Our analysis revealed that the trajectories of improvement or worsening change over time, and our statistical program was able to detect the break points where the curve changed, which in all cases was a leveling-off of the initial trajectory. Segment breakpoints for several sub-group analyses of the study population are presented in Table 2. The largest factor determining both direction of the trajectory and the rapidity of stabilization was ventilator status, with non-ventilated patients stabilizing, after improvement, after 7–8 days, while ventilated patients did not begin to stabilize from their downhill course until 12–13 days.

**Table 2:** Segment breakpoints in first segment coefficient by patient characteristics.

**Subgroup category**	**Segment breakpoint**	**First segment coefficient**
Overall cohort	7.4	0.102^*^
**Age**		
18–34 years old	7.6	0.171^*^
35–54 years old	6.5	0.183^*^
55–79 years old	7.5	0.081^*^
80+ years old	7.8	−0.007
**Sex**		
Males	7.5	0.092^*^
Females	7.3	0.115^*^
**Disease severity**		
Mild or moderate	6.4	0.186^*^
Severe	8.3	0.096^*^
Critical or end stage	8.4	−0.034^*^
**ICU status**		
Not in ICU	6.4	0.185^*^
In ICU	10.4	0.021^*^
**Ventilator status**		
Not on ventilator	7.4	0.143^*^
On ventilator	12.3	−0.043^*^

*All regression models were analyzed with 2 segments to assess changes and trends of the clinical outcome of each day*.

* *Indicates P < 0.001*.

### Multivariate Analysis of the Factors

To assess the individual and combined contributions of the factors described above (age, gender, ICU status, ventilator status at the time of transfusion, and a qualitative measure of baseline illness severity), we undertook a multivariate approach (Table 3), using a generalized estimating equations (GEE) model. The cumulative odds ratios from the GEE model indicate the odds for being in a higher category of the ordinal scale (net improvement or +1 on ordinal scale compared to the day that the infusion was given). Advanced age (≥80 years old) (OR = 0.26, 95% CI, 0.22–0.31, *P* < 0.0001), and critical illness upon enrollment were the strongest predictors of non-improvement following convalescent plasma treatment. ICU (OR = 0.46, 95% CI, 0.42–0.51, *P* < 0.0001) and ventilator status (OR = 0.42, 95% CI, 0.37–0.47, *P* < 0.0001) were next in importance, and gender (OR = 0.93, 95% CI, 0.86–1.01, *P* = 0.07) showed little discrimination once other factors were taken into account.

**Table 3:** Multivariate analysis of improvement trajectory based on demographics and disease severity prior to treatment with COVID-19 convalescent plasma.

**Variables**	**OR (95% CI)**	***P-*value**
**Intensive care status**		
Not in ICU prior to transfusion	1.00 (Ref)	
ICU admission prior to transfusion	0.47 (0.42–0.51)	<0.0001
**Mechanical ventilation status**		
Not on ventilator prior to transfusion	1.00 (Ref)	
On ventilator prior to transfusion	0.41 (0.37–0.46)	<0.0001
**Sex**		
Females	1.00 (Ref)	–
Males	0.93 (0.86–1.00)	0.070
**Age**		
18–34 years	1.00 (Ref)	–
35–54 years	0.83 (0.71–0.96)	0.01
55–79 years	0.48 (0.41–0.55)	<0.0001
80+ years	0.25 (0.21–0.30)	<0.0001
**Odds ratio of net improvement by day 7 stratified by disease severity**		
Mild or moderate	1.00 (Ref)	–
Severe	0.44 (0.38–0.50)	<0.0001
Critical or end stage	0.20 (0.21–0.30)	<0.0001
**Odds ratio of net improvement by day 14 stratified by disease severity**		
Mild or moderate	1.00 (Ref)	–
Severe	0.60 (0.47–0.76)	<0.0001
Critical or end stage	0.32 (0.25–0.42)	<0.0001
**Odds ratio of net improvement by day 21 stratified by disease severity**		
Mild or moderate	1.00 (Ref)	–
Severe	0.66 (0.46–0.95)	0.026
Critical or end stage	0.67 (0.46–0.99)	0.05

*Adjusted GEE model was used to estimate the odds of being in a higher category (net improvement) of the ordinal clinical outcomes. Initial status of severity and time interaction term were added in the model as time-varying covariate*.

By day 7, patients who were critical/end stage patients were 80% less likely to show improvement compared to mild/moderate cases (OR = 0.21, 95% CI, 0.17–0.25, *P* < 0.001), but by day 21, critical/end stage patients were just 40% less likely to have improved than the most favorable group (OR = 0.60, 95% CI, 0.39–0.92, *P* = 0.019). The severe illness group showed substantially less improvement than the mild/moderate group from day 7 (OR = 0.45, 95% CI, 0.39–0.52, *P* < 0.0001) to day 21 (OR = 0.65, 95% CI, 0.44–0.98, *P* = 0.039), although as with critically ill patients, the odds ratio of the severely ill did show some signs of improvement over time.

Several additional treatments were commonly used in our patient population. Azithromycin and Remdesivir were used in more than 40%, and steroids in nearly two-thirds of patients. To examine whether other medications could potentially confound the GEE model showing the categories of illness in which convalescent plasma was either more or less effective, we conducted a sensitivity analysis, adding angiotensin receptor blockers (ARBs), ACE inhibitors, azithromycin, remdesivir, steroids, and chloroquine/hydroxychloroquine into the GEE model separately. The effect sizes found for the conditions influencing convalescent plasma effectiveness changed by <10% when these medications were included in the model, indicating that our findings were not confounded by other medications.

### Discharge Status

Because some patients are discharged to hospice care, and to affirm that discharge means improvement in clinical status, we asked physicians to categorize the presumed life expectancy of discharged patients. Of the 5,521 patients in the REP without a reported death, 173 (3.1%) discharged patients were reported as “not expected to live past 30 days from date of discharge.” 4,317 (78.2%) were expected to live more than 30 days past discharge, and in 1,031 (18.7%) this status was unknown. A sensitivity analysis excluding the 173 patients who were reported as “not expected to live past 30 days from date of discharge,” showed that this exclusion did not materially alter our findings.

## Discussion

The aim of the Rapid Evaluation Project was to ascertain to what extent and how quickly COVID-19 patients improved following the transfusion of convalescent plasma. Our data describes clinical trajectories after receipt of convalescent plasma in several sub-groups of patients and may provide guidance for physicians about the best candidates to receive convalescent plasma to treat COVID-19.

We found that most patients who received COVID-19 convalescent plasma improved within the first 7–10 days following the transfusion. The trajectory of change in patients infused with convalescent plasma varied greatly depending on their disease state at the onset of treatment, and we found that some categories of patients did not appear to show any clinical benefit from convalescent plasma treatment during the first 21 days after treatment, including (a) patients who were critical or end stage at the time of enrollment, (b) patients over the age of 80, and (c) those on ventilators at the time of treatment. The latter group showed clinical deterioration following transfusion. Our results also suggest that the rate of clinical improvement in the overall cohort starts to slow down from day 7, and more noticeably after day 9 from treatment with convalescent plasma. Despite the slowing of clinical improvement after day 9, in patients not critically ill, some modest improvement continued at least until 21 days. Stability of status after the first week of treatment could also mean that convalescent plasma offers a buffer in time to allow the immune system to mount its own response to the infection and might mitigate the need for later ICU admission or mechanical ventilation. At the same time, the slowing of the rate of improvement might suggest that a second dose of passive antibody could improve outcomes if given 7–10 days after the first dose in patients whose improvement was sub-optimal (29).

Our analysis provides an estimate of effect size in risk categories while controlling for other risk factors. We found that several factors were associated with condition change following convalescent plasma treatment including: patients' level of severity at the time of treatment; ventilator use at the time of transfusion, baseline ICU admission, and age. We also observed the effect size of baseline severity changes with time which could indicate that critically ill patients need more time for the benefit of the plasma to take effect. It should, however, be noted that we cannot be certain that plasma was ineffective in slowing the progression of disease in patient groups who did not exhibit the improvement that was observed in the younger, non-ventilated patients.

These findings can illuminate the varying conclusions of meta-analyses (30). Summaries of clinical trials that are dominated by large studies in which a significant proportion of the study population were treated in ICU's and mechanically-ventilated have concluded that convalescent plasma is ineffective (31). Studies that evaluate a broader set of trials and well-controlled observational studies, and also examine sub-groups of patients treated early in the course of illness or with other markers indicating that the disease had not yet progressed to advanced illness, have found, much as we have, that convalescent plasma used early in the course of illness progression can lower COVID-19 mortality (5, 32).

### Study Limitations

Discharge of patients is not always evidence of full resolution of disease, as some patients may be discharged to hospice care. However, our findings were not altered when we excluded the small number of patients who were discharged but not expected to survive an additional month.

A second limitation is that our scale is ordinal and relative. We cannot distinguish between mild and striking improvement, or between modest and massive deterioration. However, our findings are anchored by the recording of discrete variables such as death, discharge and ventilation status daily.

Third, we provided no criteria to physicians to describe initial patient status (mild/moderate, severe, critical, end-stage) but allowed these designations to be at the discretion of the treating physician, but formal guidance for severity grading early in the pandemic was not yet well-known by US physicians. The widely-used WHO 10-point ordinal scale was published online (33) in June 2020, 3 months after the initiation of our study.

Fourth, we did not take into account other COVID-19 treatments or the timing of these treatments in relation to CP. Nearly half of the patients were treated with Azithromycin and or Remdesivir, and nearly two-thirds received steroids. Other medications were used much less frequently. It is likely that most treatments, especially steroids, were used more often in patients who were more severely ill and who we found to benefit less from convalescent plasma. It seems to us unlikely that our findings on the different effects of convalescent plasma by stage of disease were materially affected by the other medications in use.

Finally, and most critically, we have no control population untreated with convalescent plasma, and it is possible that our sub-group findings would be similar in COVID-19 patients treated with other therapies. These data cannot show that convalescent plasma is effective treatment for COVID-19. However, they clearly show that convalescent plasma is unlikely to be of much help in critically ill, end-stage patients.

Although much early use of convalescent plasma was in end-stage patients, and several large clinical trials have included mechanically-ventilated patients in their study samples, our findings point to the futility of using convalescent plasma in such circumstances. If convalescent plasma is effective, our data suggest that the beneficial effect is most likely to be found when plasma is administered early in the clinical course of illness and before the disease becomes severe.

## Conclusion

Overall, the data from our analysis provide a framework for best case uses when considering convalescent plasma to treat COVID-19. These data offer a qualitative assessment of previous analyses from our group (16) and support the conclusion that convalescent plasma maybe a valuable treatment option for some patients hospitalized for COVID-19 infection if plasma is provided early in the course of disease before patients require ventilation or are admitted to the ICU.

## Data Availability Statement

The authors are not permitted to share the data that support the findings of this study directly. Individual participant data underlying the results reported in this publication, along with a data dictionary, may be made available to approved investigators for secondary analyses following the completion of the objectives of the United States Expanded Access Program to COVID-19 convalescent plasma. Limited and de-identified data sets will be deposited into a research data repository and may be shared with investigators under controlled access procedures as approved by the Mayo Clinic Institutional Review Board. A Scientific Committee will review requests for the conduct of protocols approved or determined to be exempt by an Institutional Review Board. Requestors may be required to sign a data use agreement. Data sharing must be compliant with all applicable Mayo Clinic policies. Interested parties may contact uscovidplasma@mayo.edu or visit www.uscovidplasma.org.

## Ethics Statement

The studies involving human participants were reviewed and approved by Mayo Clinic IRB. The patients/participants provided their written informed consent to participate in this study.

## Author Contributions

TM, CW, BK, and NP contributed to conception and design of the study and wrote the first draft of the manuscript. TM, CW, AC, WG, and PJ organized the database. TM and CL performed the statistical analysis. All authors contributed to manuscript revision, read, and approved the submitted version.

## Funding

This project has been funded in part with Federal funds from the Department of Health and Human Services; Office of the Assistant Secretary for Preparedness and Response; Biomedical Advanced Research and Development Authority under Contract No. 75A50120C00096. Additionally, this study was supported in part by National Center for Advancing Translational Sciences (NCATS) grant UL1TR002377, National Institute of Diabetes and Digestive and Kidney Diseases (NIDDK) 5T32DK07352 (to CW), National Heart, Lung, and Blood Institute (NHLBI) grant 5R35HL139854 (to MJ) and grant 1F32HL154320 (to JS), Natural Sciences and Engineering Research Council of Canada (NSERC) PDF-532926-2019 (to SK), National Institute of Allergy and Infectious Disease (NIAID) grants R21 AI145356, R21AI152318 and R21 AI154927 (to DF), Schwab Charitable Fund (Eric E Schmidt, Wendy Schmidt donors), United Health Group, National Basketball Association (NBA), Millennium Pharmaceuticals, Octapharma USA, Inc., and the Mayo Clinic.

## Conflict of Interest

This study received funding from Millennium Pharmaceuticals, Octapharma USA, Inc. The funder was not involved in the study design, collection, analysis, interpretation of data, the writing of this article or the decision to submit it for publication. The authors declare that the research was conducted in the absence of any commercial or financial relationships that could be construed as a potential conflict of interest. The reviewer VB declared a shared affiliation, with the authors CW, AC, WG, JS, SK, SB, RW, and MJ to the handling editor at the time of the review.

## Publisher's Note

All claims expressed in this article are solely those of the authors and do not necessarily represent those of their affiliated organizations, or those of the publisher, the editors and the reviewers. Any product that may be evaluated in this article, or claim that may be made by its manufacturer, is not guaranteed or endorsed by the publisher.
